# Grape compounds suppress colon cancer stem cells in vitro and in a rodent model of colon carcinogenesis

**DOI:** 10.1186/s12906-016-1254-2

**Published:** 2016-08-09

**Authors:** Lavanya Reddivari, Venkata Charepalli, Sridhar Radhakrishnan, Ramakrishna Vadde, Ryan J. Elias, Joshua D. Lambert, Jairam K. P. Vanamala

**Affiliations:** 1 Department of Plant Science, The Pennsylvania State University, University Park, PA 16802 USA; 2 Department of Food Science, The Pennsylvania State University, 326 Food Science Building, University Park, PA 16803 USA; 3 The Center for Molecular Toxicology and Carcinogenesis, The Pennsylvania State University, University Park, PA 16802 USA; 4 The Pennsylvania State Hershey Cancer Institute, Penn State Milton S. Hershey Medical Center, Hershey, PA 17033 USA; 5 Center for Molecular Immunology and Infectious Diseases, The Pennsylvania State University, University Park, PA 16802 USA; 6 Department of Biotechnology & Bioinformatics, Yogi Vemana University, Kadapa, Andhra Pradesh 516216 India

**Keywords:** Colon cancer stem cells, Resveratrol, Grape seed extract, β-catenin, Chemoprevention

## Abstract

**Background:**

We have previously shown that the grape bioactive compound resveratrol (RSV) potentiates grape seed extract (GSE)-induced colon cancer cell apoptosis at physiologically relevant concentrations. However, RSV-GSE combination efficacy against colon cancer stem cells (CSCs), which play a key role in chemotherapy and radiation resistance, is not known.

**Methods:**

We tested the anti-cancer efficacy of the RSV-GSE against colon CSCs using isolated human colon CSCs in vitro and an azoxymethane-induced mouse model of colon carcinogenesis in vivo.

**Results:**

RSV-GSE suppressed tumor incidence similar to sulindac, without any gastrointestinal toxicity. Additionally, RSV-GSE treatment reduced the number of crypts containing cells with nuclear β-catenin (an indicator of colon CSCs) via induction of apoptosis. In vitro, RSV-GSE suppressed - proliferation, sphere formation, nuclear translocation of β-catenin (a critical regulator of CSC proliferation) similar to sulindac in isolated human colon CSCs. RSV-GSE, but not sulindac, suppressed downstream protein levels of Wnt/β-catenin pathway, c-Myc and cyclin D1. RSV-GSE also induced mitochondrial-mediated apoptosis in colon CSCs characterized by elevated p53, Bax/Bcl-2 ratio and cleaved PARP. Furthermore, shRNA-mediated knockdown of p53, a tumor suppressor gene, in colon CSCs did not alter efficacy of RSV-GSE.

**Conclusion:**

The suppression of Wnt/β-catenin signaling and elevated mitochondrial-mediated apoptosis in colon CSCs support potential clinical testing/application of grape bioactives for colon cancer prevention and/or therapy.

**Electronic supplementary material:**

The online version of this article (doi:10.1186/s12906-016-1254-2) contains supplementary material, which is available to authorized users.

## Background

Colorectal cancer is the third most common cancer among both men and women in the United States. It is also the second most common cause of cancer-related deaths in men and women combined [1]. With regular screening, colon cancer can be detected early, when treatment is most effective; however, in the majority of cases colon cancer is detected late. Over 95 % of colon cancer cases are considered sporadic thus placing environmental factors as the major cause [1]. The most important environmental factors among them are diet and lifestyle. The highest incidence rates of colon cancer are in developed nations including the U.S. [2]. Diets rich in refined starch, sugar, and saturated and trans-fatty acids but poor in fruits, vegetables and whole grains (prevalent in developed nations), have been shown to be closely associated with an increased risk of colon cancer [2–4]. A meta-analysis of case–control studies suggests that fruit consumption was associated with a 13 % decrease in colon cancer risk. The benefits from consuming a diet rich in fruits and vegetables could be attributed to the plethora of bioactive compounds present in them [5].

Grapes are consumed around the world and are a rich source of many bioactive compounds. Red grapes are rich in resveratrol (RSV), a stilbene that has shown anti-cancer properties in a variety of models, including human studies [6]. We previously reported that RSV suppressed proliferation and induced apoptosis via p53 activation in HT-29 and SW-480 human colon cancer cell lines, however, it was effective only at higher concentrations (75–100 μM) [7]. Grape seed extract (GSE) is a popular dietary supplement rich in proanthocyanidins and has been reported to have anti-colon cancer properties in a variety of in vitro and in vivo models [8]. As bioactive compounds exist in a complex mixture in fruits and vegetables, laboratory assessment of their biological activity in combination is more relevant to human exposure. In addition, because these compounds have pleiotropic effects, there is the potential that they will exert additive or synergistic chemopreventive actions. A recent study that compared GSE induced anti-cancer effects to the effects of its individual components found that GSE was more potent in growth inhibition compared to its individual constituents epigallocatechin, procyanidins and their association [9]. Our previous studies also support this notion, as we demonstrated using a well-established combination index method that a RSV (~25 μM) and GSE (35–50 μg/ml) mixture was potent in suppressing proliferation and elevating apoptosis in the HCT-116 human colon cancer cell line at lower concentrations compared to RSV or GSE alone [10, 11]. Combination index methods is based on the classic isobologram equation CI = D1/d1 + D2/d2. D1 and D2 are the doses of RSV and GSE respectively in the combination system where as d1 and d2 are the doses of RSV and GSE alone for the same fractional inhibition, respectively. In addition, RSV potentiated GSE-induced p53-dependent apoptosis via mitochondrial apoptotic signaling, and demonstrated specificity to cancer cells, as it was non-toxic in the normal colonic epithelial cell line CRL-1831 [10]. Our preliminary results led us to believe that a combinatorial approach towards colon cancer chemoprevention using bioactive compounds is a feasible strategy.

Historically, colon tumorigenesis has been viewed as a stochastic model where wide populations of abnormal colonocytes have an equal propensity to initiate tumor growth [12]. However, the cancer stem cell (CSC) theory suggests that most, if not all, cancerous tumors are driven by CSCs, probably through dysregulation of self-renewal pathways [13]. CSCs are capable of self-renewal, cellular differentiation, and maintain their stem cell-like characteristics even after invasion and metastasis [14]. Furthermore, they are resistant to standard therapies and thus are thought to be responsible for cancer relapse. The Wnt/β-catenin signaling pathway plays a critical role in maintenance of stemness, and survival/proliferation of CSCs [15], and as such, targeting the Wnt/β-catenin signaling is a good strategy for cancer prevention. Aberrant Wnt signaling in colon cancer is typically followed by mutation in the K-ras gene and loss of the tumor suppressor p53. It is estimated that p53 is abnormal in 50 to 75 % of colorectal cancer cases, and that this change marks the transition from noninvasive to invasive disease [16, 17]. Thus, treatments, which act independent of p53 status, are desirable.

Sulindac, a nonsteroidal anti-inflammatory drug has shown promising results in treatment of colon cancer. Recently it was shown to induce SMAC (a mitochondrial apoptogenic protein)-dependent apoptosis in cells with nuclear β-catenin (an indicator of colon CSCs) and decrease polyp numbers in the *APC*^*Min/+*^ mouse that is highly susceptible to spontaneous intestinal adenoma formation [18]. Chemotherapeutic drugs like sulindac are effective against certain types of cancers but can also have unexpected adverse effects such as gastrointestinal bleeding, hepatotoxicity [19, 20] and in some cases chronic inflammation promoted colon cancer [21]. This has stimulated active pursuit of new approaches and/or combination strategies for cancer chemoprevention. Based on our preliminary data, we hypothesized that the combination of RSV and GSE suppresses proliferation and induces apoptosis in colon CSCs. Azoxymethane (AOM), a DNA alkylating agent, induced mouse colon cancer model is a well-established and reproducible model of sporadic colon carcinogenesis to predict chemopreventive efficacy [22]. Intraperitoneal injection of AOM in A/J mice, a breed that is susceptible to chemically-induced carcinogenesis, for six weeks resulted in tumor formation within six weeks of the last injection [23]. Thus, we determined the efficacy of the RSV-GSE combination using A/J mice with six week AOM injection regimen and compared its effects to those of sulindac. Furthermore, we examined the possible molecular mechanisms that underlie the anti-cancer activity of RSV-GSE (and compared to sulindac) using colon CSCs, positive for CD 44, CD 133 and ALDH1b1 markers, isolated from primary human colon cancer tumors.

## Methods

### Chemicals

Grape seed extract (GSE, ORAC value 9000–13,000 μmole Trolox equivalents/g, total phenolic content > 85 % gallic acid equivalents) was a generous gift from San Joaquin Valley Concentrates (Fresno, CA). We had previously characterized the GSE used in this study using UPLC-MS and we detected presence of (+)-catechin and (−)-epicatechin monomers and their oligomers, and their gallate derivatives similar to other published papers [24, 25]. The GSE used in this study lacks resveratrol (RSV) as described earlier [10]. BrdU Cell Proliferation Assay Kit was obtained from Cell Signaling Technology (Danvers, MA). Antibodies for PARP and cleaved PARP, p53, pGSK3β, Bax, Bcl-2, β-actin, β-catenin, cyclin D1, c-Myc, COX-2 and topoisomerase-2β (Topo ii b) were purchased from Santa Cruz Biotechnology (Santa Cruz, CA). Cytochrome C was obtained from Cell Signaling Technology (Beverly, MA). All other chemicals including RSV were obtained from Sigma (St. Louis, MO).

### Animal study

A/J male mice (six weeks old; *n* = 13 per group) purchased from the Jackson Laboratories (Bar Harbor, ME) were housed in stainless steel wire cages (three or four per cage) with a 12 h light/dark cycle. Mice were allowed access to laboratory rodent chow and water ad libitum. After two weeks of acclimatization, all mice were randomly assigned to four groups and fed AIN-93G diets obtained from Harlan Laboratories (Indianapolis, IN).

### Azoxymethane carcinogen injection

All mice except saline controls received six weekly subcutaneous injections of azoxymethane (AOM, Sigma) in saline for colon carcinogenesis at 5 mg/kg starting at eight weeks of age.

### Experimental diets

At 16 weeks of age i.e., two weeks following the last AOM injection, the animals were assigned to the following diets – AIN-93G control, AIN-93G supplemented with RSV-GSE (0.03 and 0.12 % w/w, respectively) or AIN-93G supplemented with sulindac (0.06 % w/w). RSV and GSE concentrations were chosen based on the earlier human (*n* = 32) study that showed a decrease in serum oxidative stress markers in obese subjects orally supplemented with RSV and GSE separately [26]. Sulindac concentration was chosen based on previous clinical trial study in humans (*n* = 12) with familial adenomatous polyposis where administration of sulindac resulted in significant reduction of polyp number [27]. The saline control animals received AIN-93G control diets. All animals had free access to food and water.

### Colon tissue collection

After one week of dietary intervention, five animals from each group were euthanized using isoflurane. The remaining animals (*n* = 8/group) were euthanized after four weeks of dietary intervention. The colon was resected and washed with RNAse free PBS and observed under a dissection microscope for counting tumors. Tumor number was recorded for each animal. At the end of the study the tumor number was averaged for each treatment group and represented as means ± S.D. For immunohistochemistry and immunofluorescence analysis, about 1 cm of the colon tissue was collected and fixed with 10 % buffered formalin. Specimens were then paraffin-embedded and orthogonally sectioned. The tissue was sectioned at four microns thickness and mounted on positively charged slides.

### Immunofluorescence staining

#### Pre-treatment of slides

Prior to staining, the paraffin was softened and the tissue specimens fixed additionally by baking the slides in an oven at 55 °C for 20 min. Deparaffinization was performed with Fisherbrand (Pittsburg, PA) clearing agent citrisolv twice for five minutes and hydrated with decreasing concentrations of ethanol (100-100-95-70 v/v). For target retrieval, the slides were incubated in citrate buffer at pH 6 (9 mM citrate, 1 mM citric acid) at 95 °C for 20 min. To quench auto fluorescence from formalin residues, slides were pretreated with sodium borohydride (1 mg/mL) for five minutes. Mouse sections were blocked with mouse IgG serum from the M.O.M kit and avidin/biotin obtained from Vector Labs (Burlingame, CA) as per the manufacturer’s protocol.

#### β-catenin staining

β-catenin staining was performed at 4 °C overnight using a Abcam rabbit anti-β-catenin antibody (Cambridge, MA). Biotinylated secondary antibody in combination with streptavidin fluorescein (Vector Labs) was used for visualization. Mounting media with DAPI (Vector Labs) was used as a counterstain. All images were taken in Olympus BX-63 microscope with the help of Cell Sens software from Olympus America (Center Valley, PA).

#### TUNEL staining

TUNEL staining was performed using a cell death detection kit from Roche Diagnostics (Indianapolis, IN) according to the manufacturer’s protocol for formalin fixed paraffin embedded tissues.

### Cancer stem cells

Isolated human colon CSCs positive for cancer stem cell markers CD133, CD44, CD34, aldehyde dehydrogenase, telomerase, Sox2, cKit, and Lin28, were obtained from Celprogen Inc. (San Pedro, CA). To maintain the cells in their undifferentiated state, colon CSCs maintenance media and specially coated cell culture flasks obtained from Celprogen were used. Cells were maintained in incubation at 37 °C and 5 % CO_2_. Cell cultures at approximately 80 % confluence were used for all in vitro experimental procedures. For all experiments low passage number (less than 10) cells were used (not more than three weeks after resuscitation). The authentication information for the cell line obtained from Celprogen is available under supplemental information.

### Lentiviral shRNA-mediated attenuation of p53 in colon CSCs

Lentiviral particles encoding shRNA targeting p53 obtained from Santa Cruz Biotechnology were used to attenuate p53 expression in colon CSCs as described earlier [28].

### Cell proliferation

Cell viability was assessed by BrdU (5-bromo-2′-deoxyuridine) assay kit from Cell Signaling Technology (Danvers, MA). Briefly, cells were plated at a density of 1 × 10^5^ per well in 12-well plates. Media was replaced after 24 h with serum-free colon CSCs media (Celprogen) and dosed with RSV-GSE and/or sulindac. For all in vitro experiments sulindac sulfide, the active form of sulindac was used. Preliminary experiments revealed that lower concentrations of RSV-GSE were potent in suppressing proliferation of colon CSCs compared to the concentrations used in our earlier study using HCT-116 early colon cancer cells [10]. Interestingly, other researchers also showed that dietary bioactive compounds are more potent against highly proliferating or advanced cancer cells that are distinctly different from normal cells [29]. Hence, for this study doses of RSV were kept constant at 9 μM, whereas GSE doses in the combination varied (6.25, 12.5 and 25 μg/mL). Sulindac was dosed at 6.25, 12.5 and 25 μg/mL. After 24 h, BrdU incorporation was assayed as described in manufacturer’s protocol. The experiment was carried out in triplicate, and results were expressed as the means ± S.E.

### TUNEL assay

Apoptosis was quantified by using fluorescein labeled nucleotide and terminal deoxynucleotidyl transferase (TdT) to identify DNA fragmentation (characteristic of apoptosis). Briefly, cells (9 × 10^4^) were seeded in four-chambered glass slides, and after treatment with RSV-GSE or sulindac for 12 h, the in situ cell death detection kit from Roche Diagnostics was used for quantifying apoptosis based on the manufacturer protocol. The experiment was carried out in triplicate, and results were expressed as means ± S.E.

### Sphere formation assay

Briefly, colon CSCs (10,000 cells per well) were cultured in stem cell specific serum free media in an ultra-low attachment six-well plates. The cells were maintained in similar conditions as mentioned earlier under the cancer stem cells section. RSV-GSE or sulindac was added six hours after the cells were added to the six-well plates. At the end of ten days, the number of spheres was assayed using a phase contrast microscope.

### Western blot

Cells were plated in six-well plates at a concentration of 3.0 × 10^5^ cells per well in colon CSCs media. After 24 h, cells were transferred to serum free medium for 18 h. Protein was extracted according to our previously published protocols [10, 30]. The blots were incubated with primary antibodies overnight at 4 °C at a dilution of 1:500. Subsequently, the blots were incubated with secondary antibodies for two hours at room temperature at a dilution of 1:10,000. Blots were imaged and quantified using the Odyssey infrared imaging system and software (Lincoln, NE) and normalized to β-actin, a loading control for cytoplasmic proteins and topoisomerase-2β as a loading control for nuclear proteins. Each treatment was carried out in triplicate, and results were expressed as means ± S.E.

### Statistical analysis

Data are expressed as means ± S.E. for all the data. Significance was determined by one-way ANOVA with post hoc Tukey analysis for in vitro data (SPSS v21, IBM, Armonk, NY). For animal studies, analysis of data was done using mixed procedure in SAS v9.4 software (Cary, NC). The *p* values < 0.05 were considered statistically significant.

## Results

### RSV-GSE suppressed AOM-induced tumor incidence in mice

**Fig. 1:**
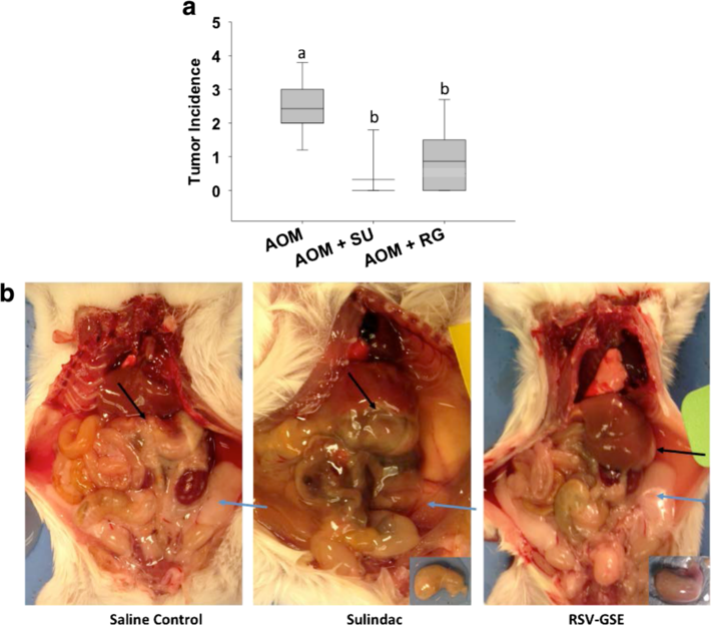
RSV – GSE suppressed tumor incidence in the colon similar to that of sulindac. **a** Mice injected with AOM consumed control, RSV-GSE or sulindac (positive control) supplemented diet for four weeks and were euthanized. Whole colon tissue was resected and observed under a dissection microscope for visible tumors. SU = Sulindac; RG = RSV-GSE. Values are in means ± S.E. (*n* = 8 in each group). Means that differ by a common letter (*a*, *b*) differ at *p* < 0.05. **b** Short-term feeding of sulindac resulted in stomach ulcers (hyperplasia of the stomach, *black arrows*) and loss of adipose tissue deposits (*blue arrows*) compared to control. RSV-GSE supplemented diet consuming animals showed neither hyperplasia nor loss of adipose tissue deposits

Mice exposed to AOM developed colon tumors at the end of the study. The incidence of AOM-induced tumors was suppressed in the RSV-GSE group by over 50 % (Fig. 1a), an effect similar to that of sulindac. Sulindac treatment resulted in significant gastrointestinal toxicity (stomach/intestinal ulcers) marked with loss of fat deposits (Fig. 1b). Such toxicity was not observed in the animals consuming RSV-GSE. Neither RSV-GSE nor sulindac significantly affected average weight gain or food intake across the groups.

### RSV-GSE induced apoptosis and reduced number of crypts with colon cancer stem cells

**Fig. 2:**
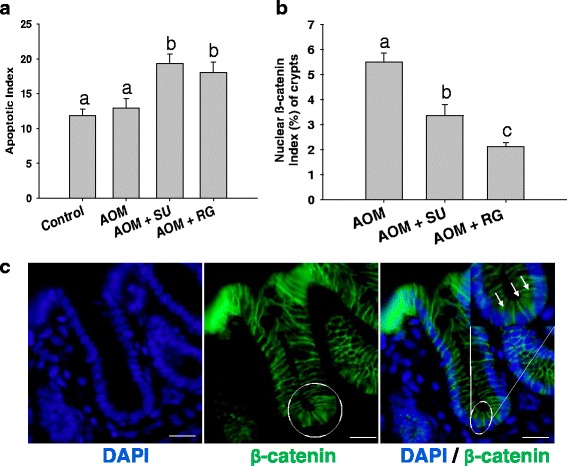
RSV – GSE treatment induced apoptosis and reduced the number of crypts containing cells with nuclear β-catenin (an indicator of colon CSCs). Mice injected with AOM were fed with control, RSV-GSE or sulindac-containing diet for one week. Distal colon sections from the mice were analyzed for TUNEL positive crypts and β-catenin localization by immunofluorescence. **a** The fractions of crypts containing at least one TUNEL-positive cell (indicator of apoptotic cells) were determined. **b** Quantification of crypts with nuclear β-catenin in mice treated with control, RSV-GSE or sulindac supplemented diet for one week. Accumulation of nuclear β-catenin is hallmark of cancer stem cells and hence was used as an indirect measure for evaluating elimination of cancer stem cells. **c** Staining of β-catenin and DAPI (*blue*) in mice treated with AOM. Circles mark representative colon stem cells with nuclear β-catenin (CSCs). SU = Sulindac; RG = RSV-GSE. Values are in means ± S.E. (*n* = 5 in each group). At least 300 crypts from each animal were analyzed. Means that differ by a common letter (*a*, *b*, *c*) differ at *p* < 0.05. (Scale bars: 15 μm)

Previously conducted studies determined a one week time window for analyzing sulindac-induced apoptosis in intestinal stem cells in *APC*^*Min/+*^ mice because of the rapid and transient nature of apoptotic events. Here we found that RSV-GSE supplementation for one week induced apoptosis with 18 % of crypts containing at least one TUNEL-positive cell, an effect comparable to the 18.5 % in mice receiving sulindac (Fig. 2a). In addition, RSV-GSE and sulindac treatment for one week also reduced the number of crypts containing cells with nuclear β-catenin (an indicator of colon CSCs) by more than 50 % (Fig. 2b, c). These data demonstrate that intestinal stem cells with nuclear β-catenin (CSCs) may be targeted for apoptosis induction following RSV-GSE or sulindac treatment in mice with AOM induced colon carcinogenesis. This might also explain lower tumor incidence in the RSV-GSE and sulindac groups at the end of the study (Fig. 1a).

### RSV-GSE suppressed proliferation and induced apoptosis in colon cancer stem cells

**Fig. 3:**
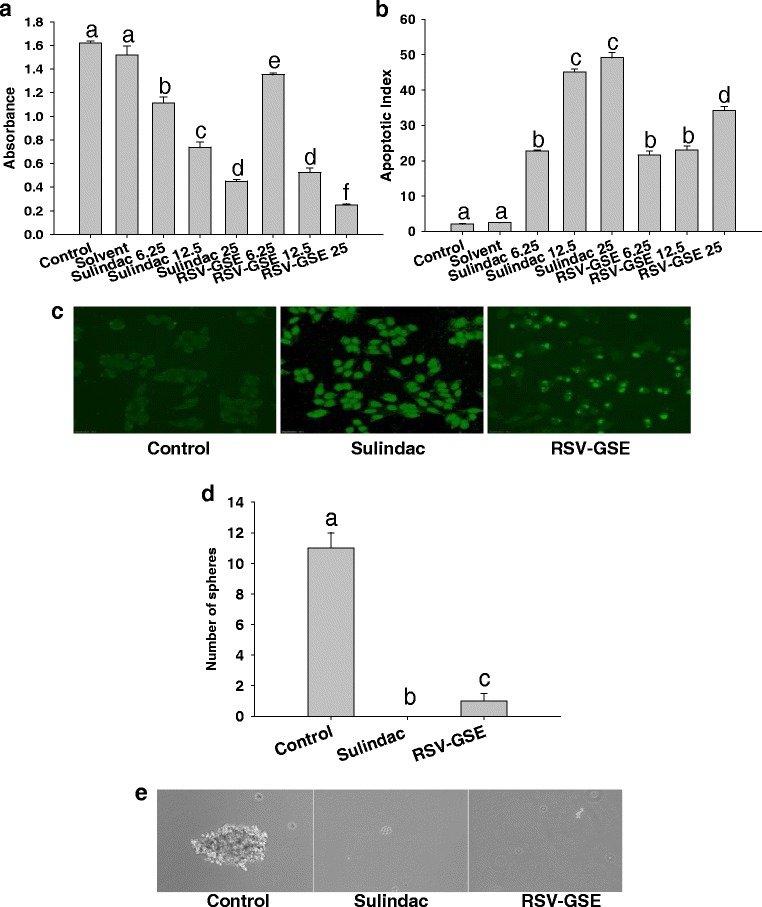
RSV – GSE suppressed proliferation, induced apoptosis and suppressed sphere formation in colon CSCs similar to that of sulindac. **a** Anti-proliferative effect of RSV-GSE in colon CSCs. RSV-GSE induced apoptosis in CSCs (**b**, **c**) similar to that of sulindac. CSCs were treated with sulindac (6.25, 12.5 and 25 μg/mL) or RSV-GSE (RSV - 9 μM and GSE 6.25, 12.5 and 25 μg/mL) for 24 h and BrdU assay was performed to assess proliferation. TUNEL assay was performed based on manufacturer protocol (Roche) and the results are expressed as per cent apoptosis. Cells fluorescing bright green due to fragmented DNA indicate apoptotic cells. Pictures taken on fluorescence microscope at 20× magnification. Representative pictures are shown for Control, RSV-GSE at 9 μM and 12.5 μg/mL respectively and sulindac at 12.5 μg/mL. **d**-**e**, Sphere formation was assessed as described in methods. Representative images taken from the sphere formation assay are presented. Results were expressed as mean ± S.E. for three experiments at each time point. Means that differ by a common letter (*a*, *b*, *c*, *d*, *e*, *f*) differ at *p* < 0.05

Proliferation and apoptotic response were determined in isolated human colon CSCs in response to RSV-GSE or sulindac treatment using BrdU incorporation and TUNEL, respectively. Both RSV-GSE and sulindac induced dose-dependent suppression of cell proliferation (Fig. 3a) and elevated apoptosis (Fig. 3b, c) in colon CSCs. The IC(50) values for RSV-GSE was determined to be 9 μM and 12.5 μg/mL respectively, and for sulindac at 12.5 μg/mL which are at physiologically relevant doses. Thus, we used these doses for subsequent experiments to determine the mechanism of action.

### RSV-GSE suppressed sphere formation ability of colon CSCs

To assess RSV-GSE ability to target the self-renewal capability of CSCs, sphere formation assay was used (Fig. 3d). Figure 3d shows representative images collected from the sphere formation assay and demonstrate the decreased number of spheres associated with the treatments in comparison to the control. RSV-GSE treatment completely suppressed colon CSCs sphere formation. This demonstrates that, in addition to the anti-proliferative and pro-apoptotic activities, RSV-GSE alters the stem-like properties by inhibiting colon cancer stem cell self-renewal as measured using the sphere formation assay.

### RSV-GSE suppressed Wnt pathway proteins

**Fig. 4:**
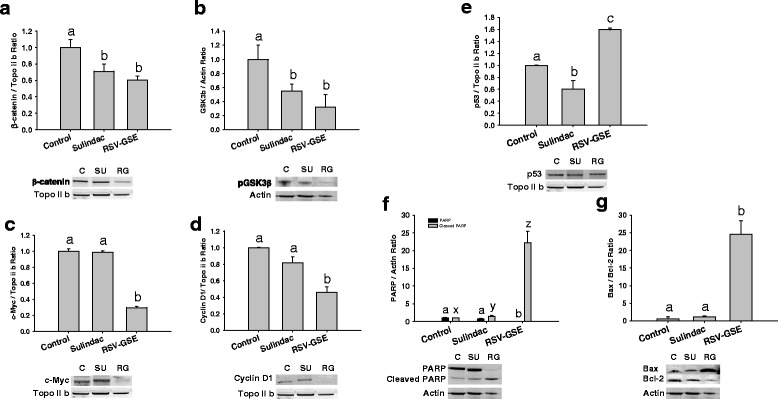
**a**–**d**, RSV – GSE suppressed levels of proteins involved in Wnt/β-catenin pathway in colon CSCs with functioning p53. Nuclear β-catenin (**a**) and its regulator phosphorylated GSK3β (**b**) levels were suppressed by RSV-GSE similar to that of sulindac. Downstream targets of Wnt/β-catenin pathway – c-Myc (**c**) and Cyclin D1 (**d**), in the nucleus were suppressed by RSV-GSE compared to sulindac. E-G, RSV-GSE induced apoptosis via p53 dependent pathway in colon cancer stem cells (CSCs) with functioning p53. Nuclear p53 levels were elevated (**e**) by RSV-GSE but not sulindac. Cleaved PARP (**f**) and Bax/Bcl-2 ratio (**g**) were also elevated by RSV-GSE but not sulindac. Colon CSCs were treated with RSV-GSE at 9 μM and 12.5 μg/mL, or sulindac at 12.5 μg/mL for 24 h, and cytosolic and nuclear cell lysates were analyzed for respective proteins by western blotting. Actin and topoisomerase-2β (Topo II b) were used as loading controls for cytosolic and nuclear proteins respectively. Values are in means ± S.E. Means that differ by a common letter (*a*, *b*, *c*, or *x*, *y*, *z*) differ at *p* < 0.05

As the Wnt/β-catenin signaling pathway is critical for stem cell fate, we treated colon CSCs with RSV-GSE or sulindac and measured proteins in the pathway - pGSK3β (cytoplasmic) and, β-catenin, c-Myc and cyclin D1 (all nuclear) using western blotting. Both RSV-GSE and sulindac treatment suppressed protein levels of pGSK3β in the cytoplasm and nuclear levels of β-catenin. This indicates reduced translocation of β-catenin to the nucleus and thus suppression of the canonical Wnt/β-catenin signaling that is frequently deregulated in colon cancer (Fig. 4a, b). Downstream proteins of β-catenin, c-Myc and cyclin D1, critical in stem cell proliferation, were also suppressed by RSV-GSE treatment. However, sulindac treatment failed to induce any changes in c-Myc and cyclin D1 levels (Fig. 4c, d).

### RSV-GSE elevated mitochondrial apoptotic pathway proteins

P53 is a critical transcription factor that controls cell fate in response to various stresses. In addition, as “the guardian of the genome”, p53 protein plays a critical role in tumor suppression by inducing growth arrest, apoptosis, and senescence, as well as by blocking angiogenesis. Nuclear levels of p53 were elevated by RSV-GSE treatment, but not sulindac, compared to control in colon CSCs (Fig. 4e). Downstream of p53, Bax, the pro-apoptotic protein was elevated and Bcl-2, the anti-apoptotic protein, was suppressed by RSV-GSE treatment indicating mitochondrial-mediated apoptosis. Bax/Bcl-2 ratio was elevated only in the RSV-GSE group but not sulindac compared to the control (Fig. 4g) in colon CSCs. Data for cleaved PARP, indicator of apoptosis, mirrored the Bax/Bcl-2 ratio, with RSV-GSE treatment showing highest levels of cleaved PARP (Fig. 4f).

### RSV-GSE efficacy is retained even in the absence of p53

**Fig. 5:**
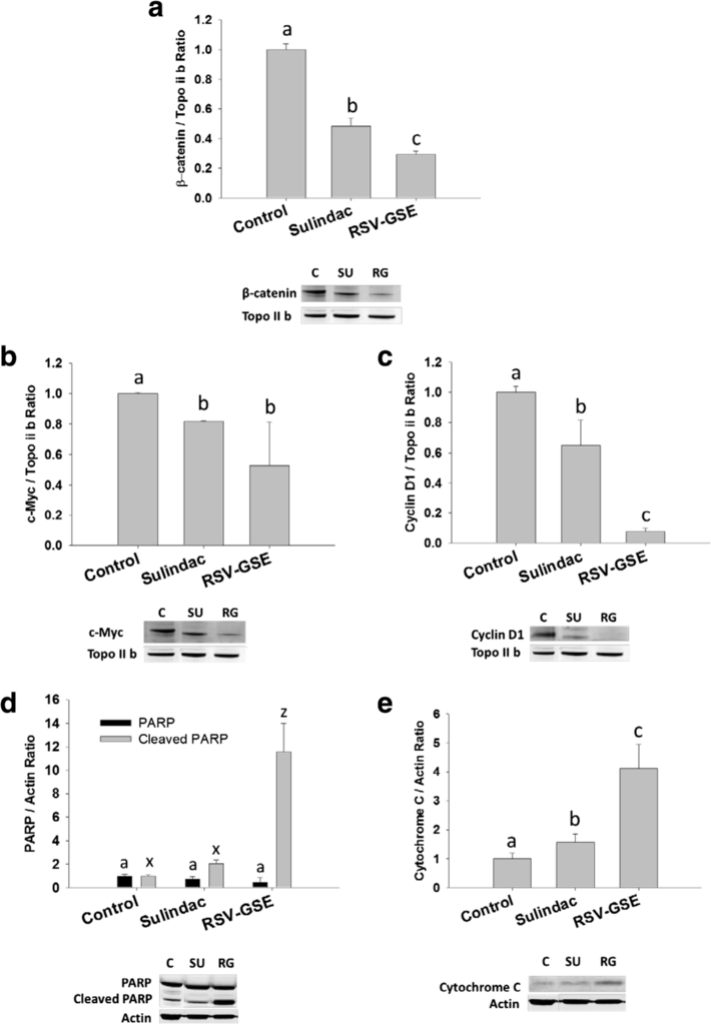
Modulation of Wnt/β-catenin and apoptotic signaling proteins by RSV – GSE in colon CSCs with attenuated p53. β-catenin (**a**) and its downstream targets c-Myc (**b**) and cyclin D1 (**c**) were suppressed by RSV-GSE compared to sulindac. Pro-apoptotic proteins cleaved PARP (**d**) and cytochrome C (**e**) levels were elevated by RSV-GSE greater than that of control and sulindac. Colon CSCs were treated with RSV-GSE at 9 μM and 12.5 μg/mL, or sulindac at 12.5 μg/mL for 24 h, and cytosolic and nuclear cell lysates were analyzed. Actin and topoisomerase-2β (Topo II b) were used as loading controls for cytosolic and nuclear proteins respectively. C = Control; SU = Sulindac; RG = RSV-GSE. Values are in means ± S.E. Means that differ by a common letter (*a*, *b*, *c*, or *x*, *y*, *z*) differ *p* < 0.05

**Fig. 6:**
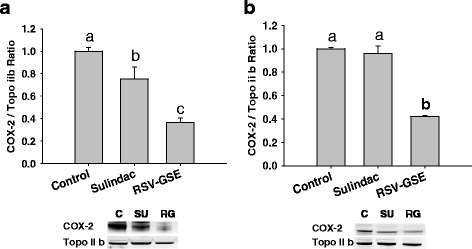
RSV – GSE suppressed COX-2 levels in colon CSCs with functioning (**a**) and attenuated p53 (**b**). Colon CSCs were treated with RSV-GSE at 9 μM and 12.5 μg/mL respectively or sulindac at 12.5 μg/ml for 24 h, and nuclear cell lysates were analyzed for COX-2 levels by western blotting. Topoisomerase-2β (Topo II b) was used as a loading control. C = Control; SU = Sulindac; RG = RSV-GSE. Values are in means ± S.E. Means that differ by a common letter (*a*, *b*, *c*) differ at *p* < 0.05

To determine the requirement of p53 in the CSC inhibitory effects of RSV-GSE, we used a lentiviral p53-shRNA construct to attenuate p53 expression. Reduced p53 expression had no effect on RSV-GSE-mediated suppression of nuclear levels of β-catenin and its downstream proteins, c-Myc and cyclin D1 (Fig. 5a–c). RSV-GSE also induced apoptosis in colon CSCs as measured using PARP cleavage (Fig. 5d) and increased cytochrome C expression greater than that of sulindac (Fig. 5e). These results indicate that GSE-RSV-induced CSC apoptosis occurs via a p53-independent mechanism. Similar trend was observed in nuclear levels of COX-2 in both colon CSCs and colon CSCs with shRNA attenuated p53 – RSV-GSE treatment was more potent in suppressing COX-2 expression compared to sulindac (Fig. 6a, b).

## Discussion

The objective of present study was to evaluate the anti-cancer efficacy of RSV-GSE in a mouse model of colon cancer, and to determine the mechanisms of action using human colon CSCs in vitro. Our results present the first evidence of in vivo anti-colon cancer efficacy of a combination of the grape bioactive components RSV and GSE in the mouse model with AOM-induced colon carcinogenesis. Our data in vitro in colon CSCs demonstrate suppression of nuclear translocation of β-catenin (Wnt/β-catenin signaling pathway) and induction of mitochondrial-mediated apoptosis.

Our results indicate that RSV-GSE, bioactive components from grapes, suppress tumor incidence in a mouse model with AOM-induced colon carcinogenesis (Fig. 1a). Furthermore, RSV-GSE consumption had reduced toxicity compared to sulindac, suggesting specific targeting of cancer cells (Fig. 1b). Indeed, clinical trials in humans have shown that RSV is quite safe [31], similar results have been observed for GSE [32].

Accumulated experimental evidence has suggested that most cancers, including colon cancer, have a hierarchal organization regulated by a small number of self-renewing cancer cells, called CSCs [33]. CSCs including colon CSCs have shown to be resistant to conventional chemotherapeutic regimens that target homogeneous populations of rapidly proliferating differentiated tumor cells. For e.g., CD133-positive colon CSCs were shown to be resistant to the conventional cytotoxic drug 5-florouracil and the resistance was shown to be dependent on Wnt signaling [34]. The proliferation and the acquisition of the stem cell fates is coordinated by a small number of highly evolutionarily conserved signaling pathways, including the Wnt/β-catenin signaling pathway, which is commonly deregulated in most colon cancers [35]. Nuclear accumulation of β-catenin is implicated in the transformation of stem cells to oncogenic stem cells in the colon [15]. Although, it has been observed that nuclear β-catenin accumulation is also seen in normal colonic stem cells and progenitor cells which are located at the bottom proliferative compartment of the intestinal crypts [36, 37], a recent study has shown that it is observed in less than 0.01 % of crypts in wild-type mice [18]. Hence, we [38] and others [18] considered increased number of crypts with colonic stem cells with nuclear β-catenin accumulation (Additional file 1: Figure S1) as a hallmark of colon carcinogenesis and a signature feature of elevated oncogenic stem cells. Qiu et al. reported that one week of sulindac treatment resulted in a 75 % reduction in the number of crypts containing cells with nuclear β-catenin. Most importantly, a vast majority (98 %) of identifiable stem cells with accumulated nuclear β-catenin in sulindac-treated *APC*^Min/+^ mice were TUNEL-positive at this time point [18]. In the current study, where AOM (a well-known colon specific carcinogen) was used to induce colon carcinogenesis, RSV-GSE consuming animals had 62 % reduction in number of crypts containing cells that have accumulated nuclear β-catenin (Fig. 2a). Additionally, our data suggest that this could be due to induction of apoptosis (Fig. 2b). Efficacy of RSV-GSE was comparable or better than sulindac. The in vivo data was supported by our in vitro observations where we noticed that RSV-GSE at physiologically relevant doses suppressed proliferation and induced apoptosis as well as suppressed sphere formation in colon CSCs (Fig. 3a–d).

There is evidence that nuclear accumulation of β-catenin results in accelerated tumor cell proliferation and tumor progression through the transcriptional activation of target genes including c-Myc, cyclin D1 and COX-2 [39]. Mechanistic data in vitro confirmed our in vivo observations as RSV-GSE suppressed nuclear β-catenin accumulation in colon CSCs (Fig. 4a). RSV-GSE also suppressed cytoplasmic levels of pGSK3β (Fig. 4b) shown to induce nuclear β-catenin translocation and down-regulated nuclear levels of proteins downstream of Wnt/β-catenin pathway, c-Myc and cyclin D1 (Fig. 4c, d). c-Myc and cyclin D1 are the key signatory genes of Wnt signaling and both function in the stimulation of cell proliferation and in preventing apoptosis. Coordination of c-Myc with cyclin D1 or its upstream activators not only accelerates tumor formation, but also may drive tumor progression to a more aggressive phenotype [40]. Although, previously published research has shown that RSV and GSE suppressed nuclear β-catenin translocation, to our knowledge, this is the first study to show such an effect in colon CSCs.

Alterations in Wnt/β-catenin signaling might also explain why RSV-GSE also suppressed sphere formation ability in vitro (Fig. 3d). Because c-Myc and cyclin D1 also play a role in stemness [41], our data showing suppressed c-Myc and cyclin D1 only by RSV-GSE treatment might explain its higher potency compared to sulindac. Our data is in line with recent research showing that dietary compounds including grape seed extract, curcumin, lycopene and resveratrol are promising chemopreventive agents against various types of cancers owing to their direct and indirect effects on CSC self-renewal pathways, such as Wnt/β-catenin signaling pathway [42–45].

P53 plays a critical role in tumor suppression by inducing growth arrest, apoptosis, and senescence, as well as by blocking angiogenesis. Consistent with the role of p53 as a cell stress-associated transcription factor [46, 47], we observed increased expression of p53 (Fig. 4e) and p53-responsive Bax (and Bax/Bcl-2 ratio) (Fig. 4g) in colon CSCs with RSV-GSE treatment. This indicates RSV-GSE induced intrinsic apoptotic signaling pathway by Bax-induced increased permeation of mitochondrial membrane, resulting in release of cytochrome C and activation of caspases. Whether similar pathway of apoptosis is activated in p53 knockout cells remains to be seen, although cytochrome C was elevated by RSV-GSE treatment in colon CSCs with shRNA attenuated p53. Mutational inactivation of p53 is one of the most frequent events found in over 50–75 % of colon cancer cases, and marks transition to metastasis [17, 48–50]. Our results showing that RSV-GSE exerts its biological efficacy, both anti-proliferative and pro-apoptotic, in colon CSCs independent of their p53 status (Fig. 5a–e) confers an advantage to the use of RSV-GSE for primary and secondary chemoprevention.

Preclinical and clinical studies suggest that COX-2 is involved in chronic inflammation and its activation may be involved in inflammation-mediated stem cell proliferation/differentiation [51]. Our data suggests that RSV-GSE was more effective compared to sulindac in suppressing nuclear COX-2 levels (Fig. 6a, b) in colon CSCs and colon CSCs with shRNA attenuated p53 and might further explain higher potency of RSV-GSE combination compared to sulindac. Further, NSAIDs like sulindac can suppress both COX-1 and COX-2 thereby deplete prostaglandin in tissues, which mediate mucosal bicarbonate production, mucus secretion, and maintenance of blood flow [52] and thus mucosal healing [53]. This explains the increased gastrointestinal toxicity (stomach ulcers and loss of adipose tissue deposits) in mice fed with sulindac. Unlike, sulindac, RSV and GSE (proanthocyanidins) have minimal effects on COX-1/PGE2 [54] and thus explains the lack of stomach ulcers and adipose tissue loss. Thus, in the future studies, it is critical to explore whether sulindac eliminates normal colon stem cells along with colon cancer stem cells whereas RSV-GSE is selective against only colon cancer stem cells. This aspect could not be assessed in the current study, as we did not include normal control animals consuming RSV-GSE or sulindac.

## Conclusion

Our study has shown that RSV-GSE combination eliminates colon CSCs in vivo and in vitro similar to that of NSAID sulindac without any toxicity. Our results also establish the molecular basis for the beneficial effect of RSV-GSE combination that is a popular dietary supplement. Although further investigations are needed to understand more on the interactions of these agents and on long-term colon cancer chemopreventive or chemotherapeutic potential of the RSV-GSE, our findings suggest that clinical testing of RSV-GSE against colon cancer is required.

## Abbreviations

AOM, azoxymethane; CSC, cancer stem cells; GSE, Grape seed extract; RSV, Resveratrol

## Additional file

Additional file 1: Figure S1. Nuclear β-catenin localization is observed in stem cells. Co-localization of Lgr5 (green) and β-catenin (red) in mice treated with AOM. DAPI (blue) is the nuclear counterstain (panel a). Panel b shows Lgr5 positive stem cell (green) at the crypt base. Panel c shows β-catenin staining around the nucleus except at the crypt base. Panel d merged image confirms co-localization of Lgr5 and nuclear β-catenin only in oncogenic colon stem cells. Circles mark representative cell with Lgr5 and nuclear β-catenin. Scale bars: 20 μm. Formalin fixed paraffin embedded sections from three animals were analyzed and representative image(s) are shown. (PPTX 1934 kb)
