# Supplementary material for: Grape compounds suppress colon cancer stem cells in vitro and in a rodent model of colon carcinogenesis
Source: BMC Complement Altern Med. 2016 Aug 9;16:278. doi: 10.1186/s12906-016-1254-2 (PMC4977641; doi:10.1186/s12906-016-1254-2)
Supplement: Additional file 1: Figure S1. — Nuclear β-catenin localization is observed in stem cells. Co-localization of Lgr5 (green) and β-catenin (red) in mice treated with AOM. DAPI (blue) is the nuclear counterstain (panel a). Panel b shows Lgr5 positive stem cell (green) at the crypt base. Panel c shows β-catenin staining around the nucleus except at the crypt base. Panel d merged image confirms co-localization of Lgr5 and nuclear β-catenin only in oncogenic colon stem cells. Circles mark representative cell with Lgr5 and nuclear β-catenin. Scale bars: 20 μm. Formalin fixed paraffin embedded sections from three animals were analyzed and representative image(s) are shown. (PPTX 1934 kb) [file 12906_2016_1254_MOESM1_ESM.pptx]

## Slide 1
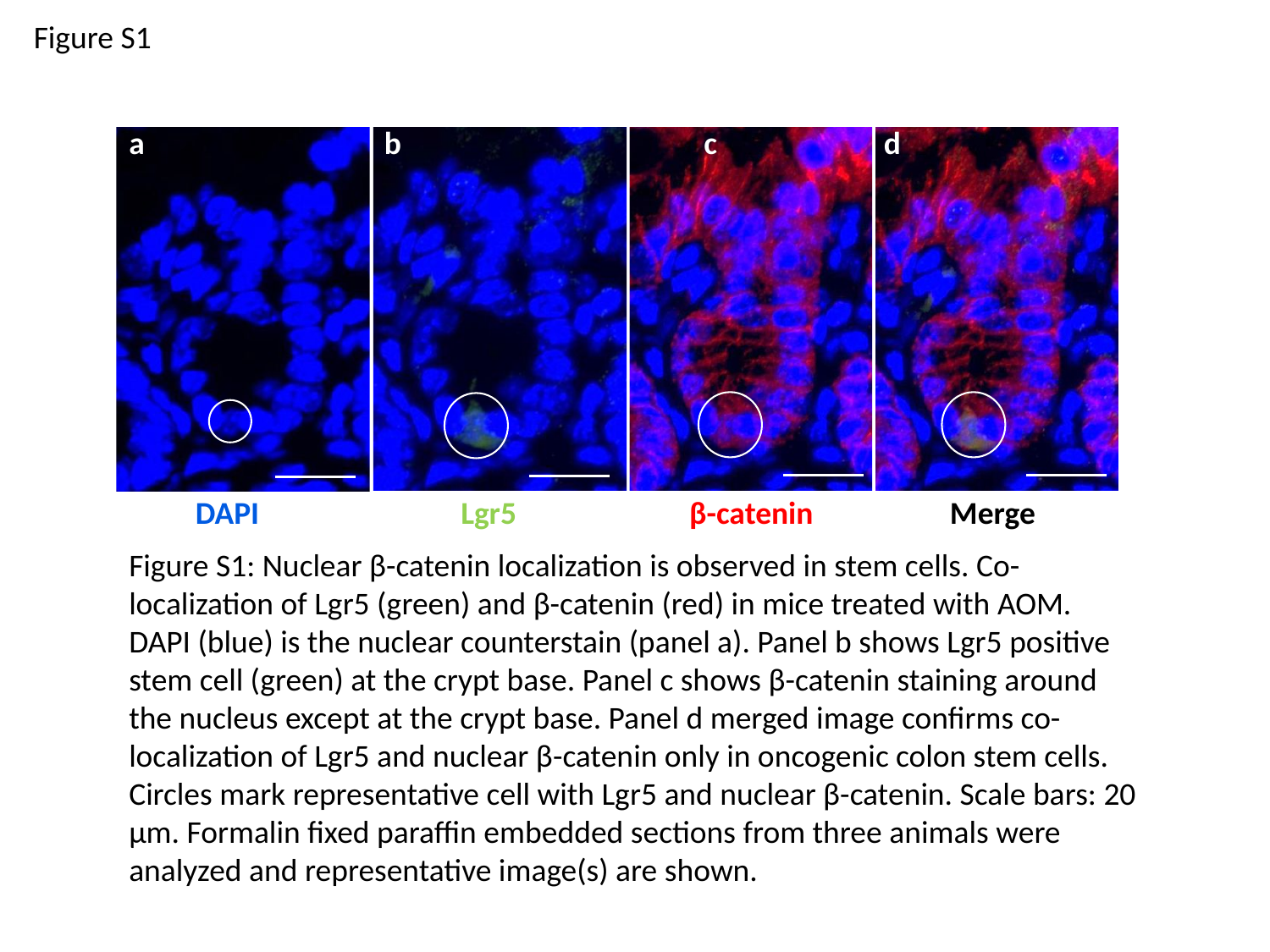

Figure S1
c
a
b
d
 DAPI Lgr5 β-catenin Merge
Figure S1: Nuclear β-catenin localization is observed in stem cells. Co-localization of Lgr5 (green) and β-catenin (red) in mice treated with AOM. DAPI (blue) is the nuclear counterstain (panel a). Panel b shows Lgr5 positive stem cell (green) at the crypt base. Panel c shows β-catenin staining around the nucleus except at the crypt base. Panel d merged image confirms co-localization of Lgr5 and nuclear β-catenin only in oncogenic colon stem cells. Circles mark representative cell with Lgr5 and nuclear β-catenin. Scale bars: 20 µm. Formalin fixed paraffin embedded sections from three animals were analyzed and representative image(s) are shown.
